# A Review on Progress, Challenges, and Prospects of Material Jetting of Copper and Tungsten

**DOI:** 10.3390/nano13162303

**Published:** 2023-08-10

**Authors:** V. Vinay K. Doddapaneni, Kijoon Lee, Havva Eda Aysal, Brian K. Paul, Somayeh Pasebani, Konstantinos A. Sierros, Chinedum E. Okwudire, Chih-hung Chang

**Affiliations:** 1 School of Chemical, Biological and Environmental Engineering, Oregon State University, Corvallis, OR 97331, USA; doddapav@oregonstate.edu; 2 School of Mechanical, Industrial and Manufacturing Engineering, Oregon State University, Corvallis, OR 97331, USA; kijoonl@umich.edu (K.L.); brian.paul@oregonstate.edu (B.K.P.); somayeh.pasebani@oregonstate.edu (S.P.); 3 Department of Mechanical Engineering, University of Michigan, Ann Arbor, MI 48109, USA; okwudire@umich.edu; 4 Department of Mechanical and Aerospace Engineering, West Virginia University, Morgantown, WV 26506, USA; hea00002@mix.wvu.edu (H.E.A.); kostas.sierros@mail.wvu.edu (K.A.S.); 5 Advanced Technology and Manufacturing Institute (ATAMI), Corvallis, OR 97330, USA

**Keywords:** inkjet printing, direct ink writing, aerosol jet printing, additive manufacturing, ink formulations, copper, tungsten, scalability

## Abstract

Copper (Cu) and tungsten (W) possess exceptional electrical and thermal conductivity properties, making them suitable candidates for applications such as interconnects and thermal conductivity enhancements. Solution-based additive manufacturing (SBAM) offers unique advantages, including patterning capabilities, cost-effectiveness, and scalability among the various methods for manufacturing Cu and W-based films and structures. In particular, SBAM material jetting techniques, such as inkjet printing (IJP), direct ink writing (DIW), and aerosol jet printing (AJP), present a promising approach for design freedom, low material wastes, and versatility as either stand-alone printers or integrated with powder bed-based metal additive manufacturing (MAM). Thus, this review summarizes recent advancements in solution-processed Cu and W, focusing on IJP, DIW, and AJP techniques. The discussion encompasses general aspects, current status, challenges, and recent research highlights. Furthermore, this paper addresses integrating material jetting techniques with powder bed-based MAM to fabricate functional alloys and multi-material structures. Finally, the factors influencing large-scale fabrication and potential prospects in this area are explored.

## 1. Introduction

Copper (Cu) and tungsten (W) transition metals have many fascinating properties. Cu has high electrical conductivity and thermal conductivity, making it a suitable candidate for printed electronics [1,2,3,4,5], catalysis [6,7,8,9,10,11,12], sensors [13,14,15], two-phase heat transfer [16], current collectors for batteries [17], solar cells [18,19,20,21,22], photo-detectors [23], and heat sinks [24,25,26]. Likewise, W also has good electrical and thermal conductivities [27], a low coefficient of thermal expansion [27], good chemical and corrosion resistance [27,28], high-temperature stability [27], and hardness, making it suitable for interconnects [29], diffusion barriers [29], solar applications [30,31,32], catalysis [33,34,35,36], armors [37,38], nuclear applications [39], and other high-temperature applications [40,41].

Due to their exceptional electrical and thermal properties, Cu and W are the primary materials of choice as interconnects, electrodes, and thermal spreaders [42,43,44,45,46,47,48,49,50]. Such applications necessitate the deposition of thin films, thick coatings, and patterning, as well as the doping of Cu or W nanomaterials into other metal matrices to produce functional composite alloys and 3D structures of Cu and W. For instance, patterned thin films of Cu/W can be utilized as gate electrodes in thin-film transistors [51,52,53,54,55] or as metal interconnects in semiconductor devices when applied as thick films [43]. Moreover, free-standing 3D structures can serve as current collectors [56] or heat sinks [57], while doping Cu or W phases into a primary metal matrix can enhance thermal properties [47,48]. Several additive and subtractive manufacturing methods, such as photolithography, plasma etching, wet etching, dry reactive etching, ion beam lithography, machining, physical vapor deposition, chemical vapor deposition, powder metallurgy, laser melting, and combinations of these methods have been used to fabricate these two transition metals or dope these materials into another material [38,48,58,59,60,61,62,63,64,65].

**Figure 1:**
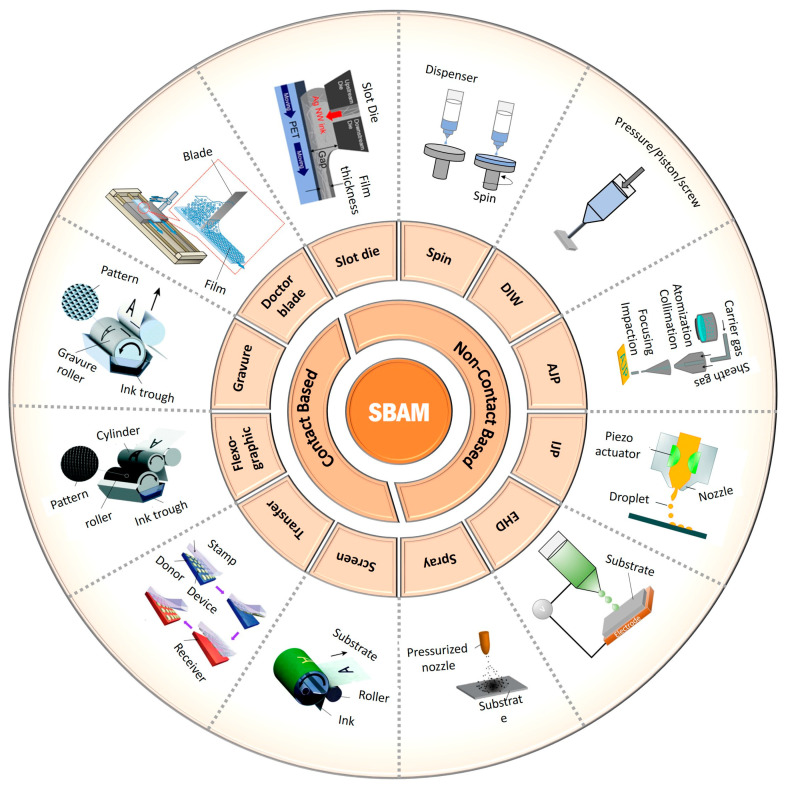
Contact and non-contact solution-based additive manufacturing techniques (schematics of printing methods adapted or reproduced with permission from [66,83,84,85,86,87,88]: copyright 2021, Elsevier Ltd.; copyright 2018, Royal Society of Chemistry; copyright 2020, Royal Society of Chemistry; Copyright 2010, American Chemical Society; copyright 2019, MDPI; copyright 2018, IOP Publishing; copyright 2016, Nature Publishing Group).

In addition to these methods, researchers have gained interest in solution-based additive manufacturing (SBAM) techniques for depositing thin films, coatings, patterning, and 3D free-form structures for various applications [66,67]. These manufacturing processes are classified as contact-based or non-contact-based, distinguished by how ink is dispensed onto the surface of choice (Figure 1). Factors such as rheological properties of ink, application type, and surface characteristics are crucial in choosing the appropriate technique for achieving good print quality [66,68,69]. Several of these methods, such as screen printing [66,70,71], transfer printing [66,72], flexographic printing [66], gravure printing [66], doctor blade coating [73], spin coating [66,74], slot-die coating, spray coating [66], inkjet printing (IJP) [66,67,75,76,77], E-jet printing (EHD) [66,78,79], aerosol jet printing (AJP) [66,80], and direct ink writing (DIW) [81,82] have been widely investigated for a diverse range of applications. The potential for scaling these processes into roll-to-roll manufacturing makes them attractive for commercialization.

This review paper focuses on non-contact printing methods, specifically material jetting technologies such as IJP, DIW, and AJP, which have the potential for integration with powder bed-based MAM. While contact-based printing methods offer some advantages, their integration with powder bed-based MAM is challenging due to the requirement for mechanical components to contact the powder bed and the difficulty of removing excess ink. In contrast, non-contact printing techniques, particularly IJP, DIW, and AJP, can be compatible with the powder bed mechanism, providing high resolution, maskless patterning, and the capability to produce thin films or three-dimensional structures. Consequently, these material jetting techniques can function as stand-alone printers for Cu and W or be integrated with powder bed-based MAM technologies, enabling voxel-level ink control. This integration facilitates the design and development of multi-material three-dimensional structures. Therefore, this review explores the progress and challenges associated with printing Cu and W using IJP, DIW, and AJP by organizing as follows: Section 2 provides a brief overview of the three material jetting printing techniques and the current research and development of these methods for various materials and applications. Section 3 offers a detailed overview of the recent progress and the challenges of Cu and W ink formulations and printing for multiple applications.

Additionally, Section 4 introduces innovative research on integrating material jetting methods with powder bed-based MAM to fabricate functional alloys and multi-material structures. Furthermore, Section 5 discusses factors that influence large-scale fabrication. Finally, the last section summarizes the article and discusses the future perspectives of SBAM and SBAM material jetting within powder bed-based MAM for Cu and W-based materials.

## 2. Material Jetting Techniques

As previously mentioned, this review concentrates on IJP, DIW, and AJP. This section briefly overviews these three printing methods and their applications.

### 2.1. Inkjet Printing (IJP)

**Figure 2:**
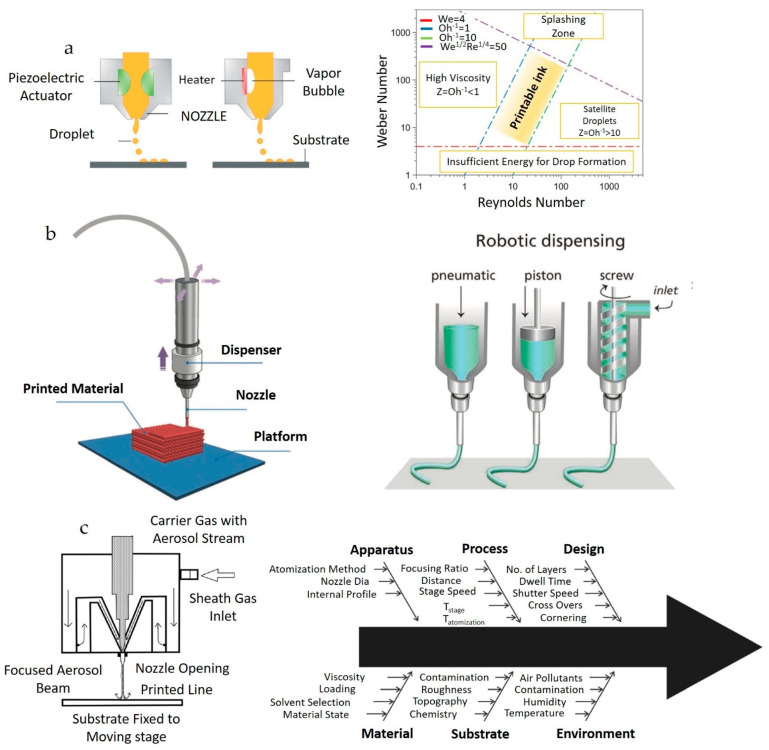
(**a**) Schematics of Piezo and thermal Inkjet printing process on the left and printability region of ink on the right (reproduced with permission from [84]: copyright 2020, Royal Society of Chemistry); (**b**) schematics of typical DIW apparatus ink on the left (reproduced with permission from [95]: copyright 2016, Royal Society of Chemistry), and different types of dispensing techniques for DIW on the right (reproduced with permission from [93]: copyright 2013, Wiley–VCH Verlag GmbH & Co., KGaA, Weinheim); (**c**) schematics of aerosol jet printing set up on the left (reproduced with permission from [98]: copyright 2013, American Chemical Society), and factors influencing the process on the right (reproduced with permission from [97]: copyright 2019, Springer).

IJP is a non-contact, additive digital printing technique used for printing patterns, sensors, electronic devices, solar cells, coatings, and 3D structures for various applications [84,89]. Since its development in the 1950s, numerous printheads have been commercialized for research and industrial purposes [90]. In this method, the materials to be printed (e.g., metal, metal oxide, organic materials) are either dissolved (precursors) or dispersed (nanomaterials) in a solvent with additives to form stable inks. Once the inks are prepared, they are filled into cartridges and printed onto the desired surface. Droplets are created by applying a voltage to piezoelectric crystals or heating thin film resistors, depending on whether a piezoelectric or thermal printhead is used, as illustrated in Figure 2a, respectively [84]. Droplet creation and stability depend on the properties (surface tension, viscosity, and density) of the inks and the printable zone, which is based on the dimensionless numbers calculated from the rheological properties depicted in Figure 2a.

### 2.2. Direct Ink Writing (DIW)

DIW is a developing AM technique used to print various materials for diverse applications [91,92]. In this robotic dispensing method, shear-thinning inks are dispensed using pneumatic, piston, or screw techniques, as illustrated in Figure 2b [93,94,95,96].

### 2.3. Aerosol Jet Printing (AJP)

AJP is an AM process that has gained significant attention due to its versatility; a relatively new technique emerged in the research field around 2001–2002 [97,98]. A collimated aerosol stream is created by generating aerosol using an ultrasonic or pneumatic atomizer and transporting the aerosol stream via a carrier gas, focusing on a sheath gas [97,98,99]. Compared to IJP, AJP offers greater flexibility, higher resolution, and a broader scope for viscosity manipulation of inks. The AJP setup and various conditions and parameters that affect the process are shown in Figure 2c.

### 2.4. Applications of IJP, DIW, and AJP

The three printing techniques discussed in the previous section are widely used to print various materials for applications in electronics, catalysis, solar cells, sensors, and batteries. Additionally, they have been employed to print active layers, dielectric layers, and gate electrodes in semiconductor devices. Recently, a broad range of materials, including metals, metal oxides, chalcogenides, perovskites, and polymers, have been printed using these methods. Table 1 summarizes the materials and techniques used for printing, along with their respective applications.

## 3. Cu and W Printing

This section aims to review various types of inks, challenges, and progress for non-contact printing of Cu and W because of their exceptional properties and resulting applications. Significant research has focused on developing Cu-based inks for conductive electronic applications. Li et al. [158] published a review paper that compiled various copper inks, including Cu nanoparticles, small molecular precursors, and mixed inks for electronic applications. In contrast, much less attention has been given to printing W nanoparticle or precursor-based inks. This result is due to the refractory metal’s extremely high sintering temperature and the seldom studied precursor chemistry for solution-based deposition systems. Table 2 provides an overview of different types of inks used and printing methods for various applications.

### 3.1. Current Reported Applications of Printed Cu and W

**Figure 3:**
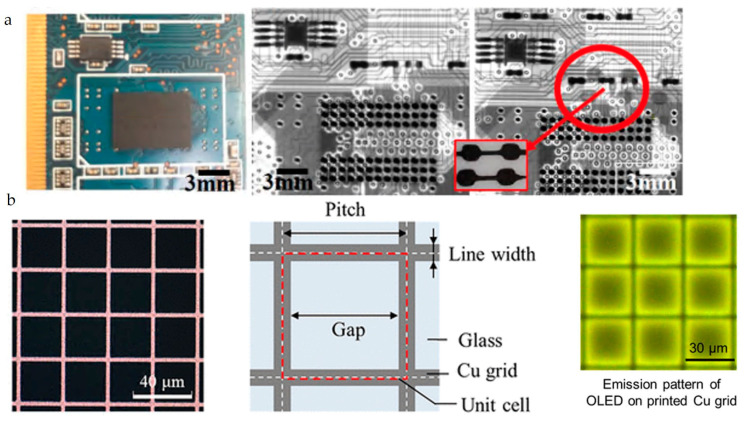
(**a**) Printed W radiation shield (reproduced with permission from [160]: copyright 2019, IOP Publishing Ltd.); (**b**) printed Cu grids for OLEDs reproduced with permission from [161]: copyright 2022, American Chemical Society).

There is limited literature on printing W for practical purposes. Recently, printed W has been used as solar absorber coatings [159] and radiation shielding on circuit boards (Figure 3a) [160]. In contrast, printed Cu has been utilized in various applications such as Cu grid electrodes for organic light-emitting diodes (OLEDs) (Figure 3b) [161], porous 3D scaffold for Li-ion batteries [56], current collecting grids for photovoltaics [162], repairing PCB boards [163], interconnects and power electronics [164,165,166], resistive temperature sensors [167]. The following section discusses the progress and challenges of printing Cu and W for these applications.

**Table 2:** Some of the works investigated Cu and W printing using different types of ink and AJP, IJP, and DIW.

Material	Ink Type	Printing Method	Post-Processing Technique	Optimum Resistivity (Conductivity)	Application	Reference
Cu	NPs	IJP	Laser sintering	0.5 µΩ □^−1^ (3.6 kS·cm^−1^)	Current collecting grids for photovoltaics	[162]
Cu	Core-shell NPS	IJP	Conventional sintering	11 µΩ·cm	Conductive patterns for electronics	[168]
Cu	NPs	IJP	Conventional sintering	13.5 µΩ·cm	Conductive patterns	[169]
Cu	Precursor	IJP	Conventional sintering	9.5 µΩ·cm	Conductive patterns	[170]
Cu	Precursor	IJP	Conventional sintering	10.5 µΩ·cm	Conductive patterns	[171]
Cu	Precursor	IJP	Conventional sintering	(15 kS·cm^−1^)	Conductive patterns	[172]
Cu	Precursor	IJP	sintering in formic acid	2.3 µΩ·cm	Conductive patterns	[173]
Cu	Precursor	IJP	Conventional sintering	-	Binding material in binder jetting, an additive manufacturing technology, to produce copper structures	[174]
Cu	NPs	IJP	Conventional sintering	-	Binding material in binder jetting, an additive manufacturing technology, to produce copper structures	[175]
Cu	NPs	IJP	Photonic sintering	<2.5 µΩ·cm	Circuits	[164]
Cu	NPs	AJP	CW laser sintering	18 µΩ·cm	To repair the PCB board	[163]
Cu–Mn	Microparticles	DIW	Conventional sintering	-	Hierarchical porous alloy could be used in catalysis, sensors, electrodes, and actuators applications	[176]
Cu	NPs	DIW	Conventional sintering	1 × 10^4^ µΩ □^−1^	Interconnects	[165]
Cu	Microparticles	DIW	Conventional sintering	(2.8 kS·cm^−1^)	Support structures for steel	[177]
Cu–Ni	NPs	AJP	Conventional sintering	1.0 *×* 10^6^ µΩ □^−1^	Resistor for power applications	[166]
Cu and alloys	NPs	AJP	Conventional sintering	-	Resistance temperature sensors	[167]
Cu	Shear thinning Cu microparticle ink	DIW	Conventional sintering	-	3D porous scaffold for Li-ion batteries	[56]
Cu	NPs	AJP	Conventional and photo sintering	<15.0 µΩ·cm	Conductive patterns	[178]
Cu and Cu-Graphene	NPs	AJP	Conventional sintering	(1.5 kS·cm^−1^)	Conductive patterns	[179]
W	Precursor	AJP	Conventional sintering	-	Porous nanostructured coating for enhancing solar absorption	[159]
W	NPs	DIW	Conventional sintering	-	Could be used for ultrahigh-voltage electric contacts	[180]
W	NPs	DIW	Conventional sintering	-	Could be used for heat exchangers	[181]

### 3.2. Commercial Cu and W Inks

The market is abundant with commercial copper nanoparticle-based inks, yet lacks metallic tungsten inks. However, there are tungsten oxide inks available, such as those provided by Sigma–Aldrich, which can be used with a reducing agent, provided the application allows for a reduction step in the procedure. The manufacturers of conductive nanoparticle inks include applied nanotechnologies, dycotech materials, novacentrix, copprint, and nanochemazone. Although the specifics of ink production largely remain proprietary information, these manufacturers typically recommend post-processing techniques, such as laser treatment, flash lamp treatment, or formic acid vapor treatment, to enhance the conductivity [182,183]. Table 3 lists some of the inks properties, carrier solvent, and processing techniques of commercial Cu inks. This table serves as a general reference; for detailed properties of a specific ink, it is recommended to refer to the corresponding manufacturer’s website.

### 3.3. Challenges and Progress of Printing Cu

Cu is an excellent conductor and can potentially replace gold (Au) and silver (Ag) as the primary component in conductive inks. These inks are in high demand in printed electronics, which seek to manufacture cost-effective, large-scale devices. While Ag is a slightly better conductor, Cu is more abundant and significantly less expensive. However, Cu is more susceptible to oxidation than Ag, a more pronounced problem for NPs. Since copper oxides act as semiconductors instead of metals, controlling oxidation is essential when employing Cu NPs in conductive inks. The primary challenge in leveraging Cu’s affordability for conductive inks is to create an economical synthesis method that yields metallic Cu NPs. Numerous innovative studies have tackled the oxidation of Cu inks during the printing, synthesis, or device fabrication process [162,168,170]. In this section, we discuss the progress made in addressing oxidation issues at different stages of the printing process and for different types of inks.

#### 3.3.1. IJP of Cu

Inkjet printing of Cu is primarily employed for printing conductive patterns for semiconductor device applications. Over the past two decades, numerous investigations have been carried out to understand substrate-ink interactions, nozzle clogging, ink rheology, the coffee ring effect, and post-treatment methods. These challenges are generally expected for any material and application. However, for metal-based inks like Cu, oxidation is the main issue. Post-processing methods like sintering in the atmosphere can lead to the oxidation of printed Cu, increasing the resistance of the patterns. Various Cu inks have been developed for different printing methods to address these challenges and study the performance of printed patterns [158].

**Figure 4:**
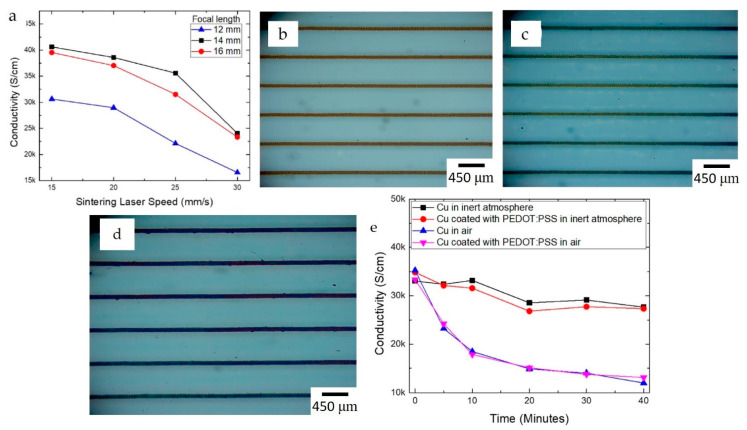
(**a**) Effect of laser sintering speed and focal length on conductivity, (**b**) printed Cu, (**c**) with 100 nm protective layer, (**d**) with 200 nm protective layer, (**e**) conductivity of Cu at different conditions (reproduced with permission from [162]: copyright 2018, Wiley–VCH Verlag GmbH & Co.).

Georgiou et al. [162] employed a fast laser sintering technique (infrared diode laser) instead of slow conventional heating to minimize the oxidization. Cu nanoparticles (NPs) (Intrinsiq Materials) dispersion ink with a viscosity of 12 cp and surface tension of 29–30 mN·m^−1^ was used to fabricate the Cu grid as a bottom electrode for solution-processed solar cells using a Fujifilm Dimatix DMP-2800 inkjet printer. An electrical conductivity of 3600 S·cm^−1^ (Figure 4a) was achieved as the optimum value without Cu layers detaching from the substrate surface. This optimal condition was obtained at a laser scanning speed of 25 mm·s^−1^ and a focal length of 14 mm. The researchers also explored doctor blade coating of Cu grids with different PEDOT: PSS layer thicknesses to protect them during solution processing of other device layers (Figure 4b–d). Despite the protective layer, sintering in ambient conditions after coating adversely affects the electrical conductivity of the Cu grid layer (Figure 4e). The authors reported that Coated Cu-based organic photovoltaics (OPV) devices showed a power conversion efficiency (PCE) of 3.35%, compared to 4.92% for ITO-based devices.

**Figure 5:**
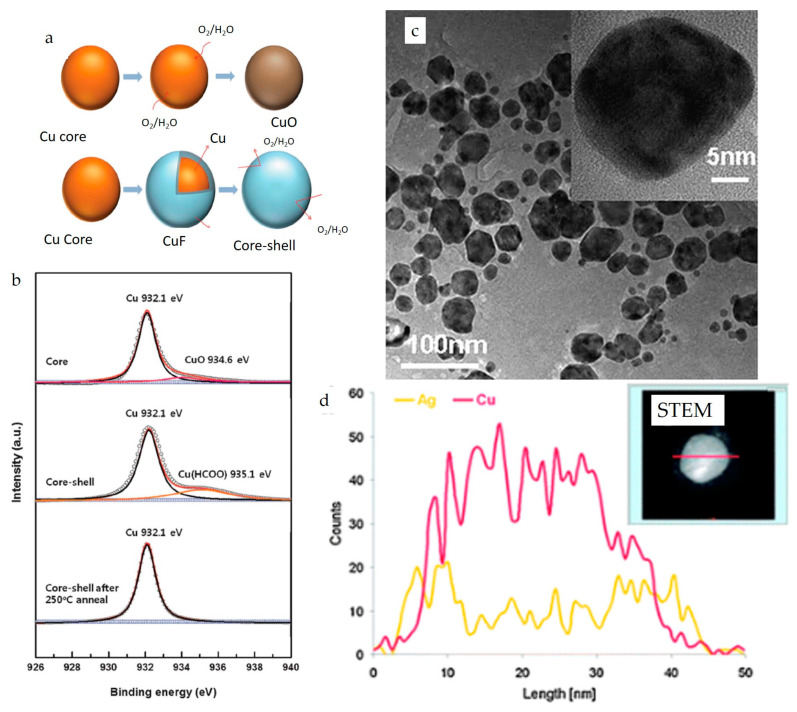
(**a**) Cu–CuF core-shell particles showing that core-shell protects the copper core from being oxidized, (**b**) XPS spectra of Cu and core-shell particles under different conditions (reproduced with permission from [169]: copyright 2013, Royal Society of Chemistry); (**c**) TEM micrograph of Cu–Ag core-shell NPs, (**d**) STEM and elemental profile along the diameter of core-shell NP (reproduced with permission from [168]: copyright 2009, Royal Society of Chemistry).

Core-shell NP inks represent another approach that has been explored to prevent the oxidation of Cu NPs and fabricate conductive Cu layers. Moon’s research group synthesized copper formate (CuF) shells around Cu to prevent oxidation, as illustrated in Figure 5a [169]. The OLA-capped Cu NPs were synthesized using copper (II) 2-ethyl hexanoate at 250 °C, and a copper formate shell was formed with the help of formic acid injection into the dispersion of 1 g of Cu NPs in acetonitrile. Subsequently, the inks were formulated by dispersing these 25 wt% core-shell NPs in 1-methoxy 2-propanol, and conductive patterns were printed using a piezo print head developed by Microfab. After annealing the films at 150 °C, the formate shell was converted to conductive copper, achieving a resistivity of 13.5 μΩ·cm. Figure 5b shows X-ray photoelectron spectra (XPS) of Cu, CuF core-shell particles, and annealed core-shell particles. It can be observed that the surface oxidation of NPs is evident from the Cu–O peak at 934.6 eV. In contrast, no oxidation is present on the annealed core-shell particles.

**Figure 6:**
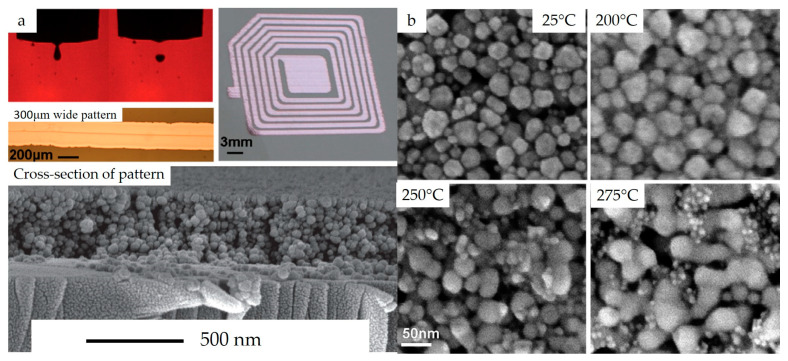
(**a**) Printed patterns and antenna using Cu-Ag core-shell NPs ink, SEM micrograph of the printed pattern, (**b**) printed patterns heat treated at various temperatures in an inert atmosphere (reproduced with permission from [168]: copyright 2009, Royal Society of Chemistry).

In another study, Grouchko et al. [168] synthesized air-stable Cu–Ag core-shell NPs (Figure 5c,d) through a two-step reaction mechanism, using copper nitrate precursor followed by the addition of silver nitrate to create a shell around Cu NPs via a transmetalation reaction. Subsequently, an ink was formulated using 25 wt% of core-shell NPs with a viscosity of 1.9 cP and surface tension of 23.9 mN·m^−1^ to demonstrate inkjet printing with the Microfab JetDrive III controller. The inks were used to print decorative and conductive patterns on various substrates, as shown in Figure 6a. The films were annealed at different temperatures to enhance their conductivity. The results indicate that the core-shell structures remained intact at 150 °C; however, above 250 °C, silver NPs were formed, and Cu NPs were no longer coated by silver (Figure 6b). Nonetheless, no further oxidation of the printed layers was observed after cooling, indicating that this ink could be useful in conductive electronics.

Additionally, numerous other studies have focused on developing core-shell NPs inks to understand the effects of oxidation. Yu et al. [184] studied the sintering behavior of Cu–Ag core-shell particles synthesized using a green approach. They proposed a sintering mechanism and found that Ag de-wetting enhanced the sintering performance and protected the Cu core from oxidation up to 156 °C. These findings highlight the need to investigate the sintering mechanism and establish a correlation between the core-shell NPs sintering mechanism and the electronic and mechanical properties of the films.

**Figure 7:**
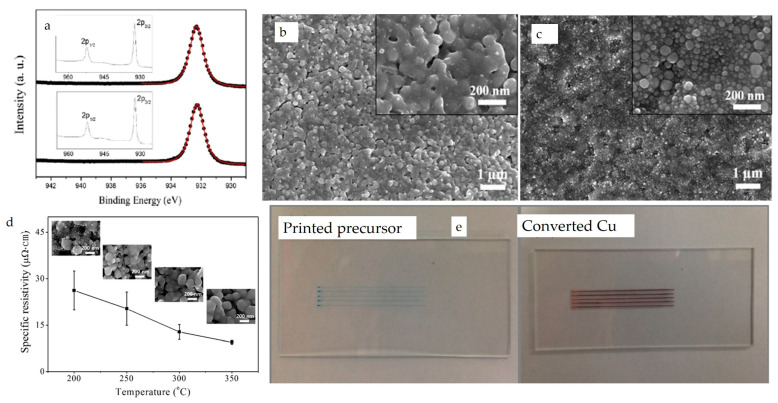
(**a**) XPS spectra of Cu films Copper formate-AMP-complex inks formed with oleic and hexanoic acids, (**b**,**c**) SEM micrographs of copper films sintered with the help of hexanoic acid and oleic acid, respectively, (**d**) resistivities of copper films at different temperatures, (**e**) inkjet printed MOD precursors (reproduced with permission from [170]: copyright 2014, American Chemical Society).

Copper molecular organic decomposition (MOD) precursors offer another approach to prevent the oxidation of Cu inks. An additional advantage of these inks is that they contain particle-free molecular precursors that can be dissolved in common solvents, such as water, alcohol, and glycol ethers for inkjet printing [170,171,172]. Shin et al. [170] used alkanolamines to modify copper formate salt and dissolved them in alcohols for printed electronics. Their research demonstrates that the 2-amino 2-methyl-1-propanol (AMP) complexation resulted in fewer carbon and oxygen impurities in the films after annealing. They also synthesized co-complexed copper formate with AMP and oleyl amine to produce more compact and dense films, as opposed to the voided films formed by the Cu formate-AMP ink. The inks were then formulated for printing by dissolving the modified precursors in alcohols after mixing them with oleic acid and hexanoic acid. The annealed Cu films showed a peak around 932.2 eV and 932.3 eV from XPS data, as shown in Figure 7a indicating that these particle-free inks are both oxidation-resistant and capable of producing Cu films without oxides. Furthermore, SEM micrographs (Figure 7b,c) show that the sintered nanoparticles connected together to form conductive copper with hexanoic acid, while small nanoparticles were generated with oleic acid. The authors attributed this to the high boiling point of oleic acid compared to hexanoic acid. The measured specific resistivities of films formed by adding hexanoic acid (which has lower resistivity compared to oleic acid) were found to be temperature-dependent, decreasing from 23.4 to 9.5 μΩ·cm when the sintering temperature increased from 200 °C to 350 °C as shown in Figure 7d. The work also demonstrated inkjet printing of MOD ink with hexanoic acid as an additive (Figure 7e). It is worth noting that another significant factor contributing to the good conductive copper films is the formation of densely packed films with minimal voids. Sintering aids have a considerable impact on the resistivities of copper films, and carboxylic acids are a class of materials that have been used for this purpose [170,173]. These carboxylic acids are miscible in most solvent systems used for Cu ink formulations.

#### 3.3.2. DIW of Cu

DIW of copper has the potential for various applications due to its excellent properties. Yet, there is limited literature on the direct writing of 3D Cu structures and ink development. When compared to the IJP process for copper, oxidation is not a significant concern in the DIW process as oxidation can be prevented through an annealing process, which is a necessary step after DIW. More importantly, a key challenge in DIW is developing a shear-thinning ink with optimal rheological properties that allow for layer-by-layer structure building.

**Figure 8:**
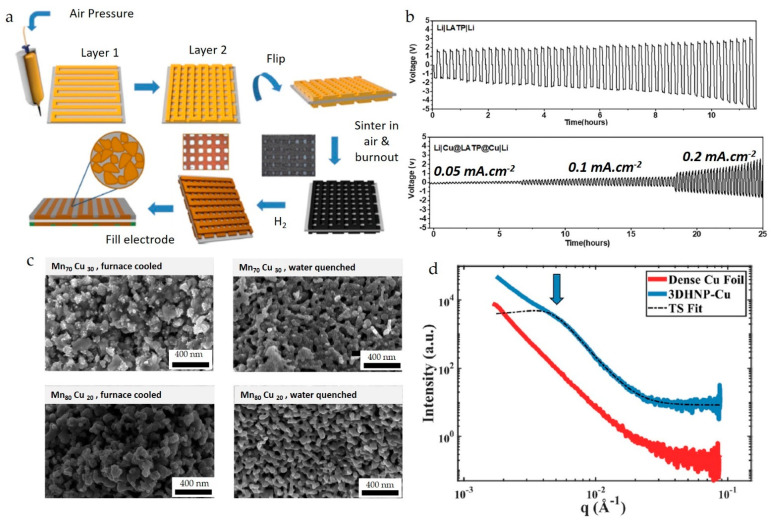
(**a**) Schematics of porous Cu grid fabricated by DIW, (**b**) voltage–time curves of Li|LATP|Li at 0.05 mA.cm^−2^ and Li|Cu@LATP@Cu|Li at three different current densities (reproduced with permission from [56]: copyright 2021, Elsevier Ltd.); (**c**) SEM micrographs of different compositions of dealloyed nanoporous 3D printed Mn_x_Cu_100-x_ at different cooling conditions, (**d**) SAXS pattern of both Cu and 3D printed Cu (reproduced with permission from [176]: copyright 2020, Elsevier Ltd.).

Research groups from West Virginia University and the University of Massachusetts, Amherst, led by Sierros and Wu, fabricated a porous Cu grid, as shown in Figure 8a, onto Li1 + xAlx3 + M2-x 4 + (PO4)3 (LATP) electrolyte for Li-ion batteries using machine learning to optimize the ink properties [56]. The ink was formulated by dispersing copper particles in a 10% (*w*/*v*) ethyl cellulose binder in ethanol, with toluene added as a humectant to prevent nozzle drying during printing. Concentrations of solids loading, solvent, and binder are crucial in any printing process for optimal printing performance. This study performs process development for ink formulations through sequential learning by examining the battery’s performance and adjusting ink parameters to attain optimal printing and battery performance. The design of experiments determined that 0.4 wt% of Cu particles, 0.56 wt% of binder solution, and 0.04 wt% humectant were the optimal parameters used to fabricate a Cu scaffold with 500 μm pores. The binder from the printed samples was removed by sintering them at 550 °C in air. The oxidized grid was reduced to metal by expositing it to the H_2_ atmosphere for 30 min. The electrochemical performance depicted in Figure 8b shows that the Li|Cu@LATP@Cu|Li significantly reduced the overpotential compared to the Li|LATP|Li cell.

DIW has also been utilized for printing 3D hierarchical nanoporous (3DHNP) Cu-based alloys, which have applications in catalysis, sensors, electrodes, and actuators [176]. Mooraj et al. [176] fabricated Mn–Cu alloy by DIW and chemical dealloying. Two different ratios of (70/30 and 80/20 at% of Mn/Cu) powders of Mn and Cu were mixed in PMMA–PnBA bi-block co-polymer binder and Tetrahydrofuran (THF) and 2-Butoxyethanol solvent mixture to formulate bi-metallic inks for printing. The printed samples were sequentially sintered to remove the solvents and polymer binder and then sintered at 948 °C and 1017 °C for Mn70Cu30 and Mn80Cu20, respectively, and cooled at different rates. Subsequently, the printed parts were dealloyed in 0.1 M HCl for 91.5 h and 4 M HCl for 12.5 h. The composition and cooling rates influenced the nanoscale morphology of the resulting 3DHNP-Cu structures, with nano-ligaments coarse and non-continuous for furnace cooled samples and smooth and continuous for water-quenched samples, as shown in Figure 8c. The Mn80Cu20 morphology, with nanoscale pores, is suitable for catalysis applications. This sample was further characterized using SAXS to determine nanoscale features, estimating the ligament-to-ligament distance of 150 nm using the Teubner-Stray model (Figure 8d). A peak at 0.005 A^−1^ was observed for 3DHNP-Cu, indicating nanostructures, and the Teubner–Strey model yielded a ligament-to-ligament distance of 150 nm.

Furthermore, DIW can be used to print patterns and circuits. However, crack and pores pose challenges in achieving high-quality printed patterns, as they can deteriorate the conductivity. Therefore, minimizing cracks and pores in printed films is crucial, as they negatively impact electrical properties. Recently, Cu NPs synthesized using the double template method were redispersed in various solvents and polymer binders to promote gel formation, which helps minimize phase separation in ink used for direct write interconnects [165]. The authors addressed these defects by optimizing solvent evaporation rates and employing double sintering to decrease cracks and pores. Consequently, they achieved a Cu film with a 0.01 Ω·sq^−1^ resistance.

#### 3.3.3. AJP of Cu

Similar to the previous section, the AJP of Cu presents challenges, such as ink formulations and post-processing techniques. Many of the ink formulations discussed in the above sections can potentially be used in aerosol jet printing systems, as these systems exhibit flexibility regarding ink rheological properties. However, care must be taken when formulating the ink, depending on the specific application of the printed materials. Despite this flexibility, factors such as print resolution, density, and post-processing techniques will influence the selection and formulation of the ink. For instance, Hilna et al. [166] investigated the printing of resistors on thick-printed copper film. Formulating inks that can be heat-treated in an inert atmosphere and do not require oxygen to remove additives from the printed film is necessary. They addressed this issue by formulating various Cu and Ni inks compositions using a mixed solvent system containing 50 wt% isopropyl alcohol, 5 wt% 2-benzyloxy ethanol, and 15 wt% 2-ethoxyethanol. Their investigation revealed that a CuNi 55:45 composition resulted in a low sheet resistance of 1 Ω·sq^−1^ and a coefficient of resistance of ±100·10^−6^ K^−1^, making it suitable for shunt resistors. Furthermore, previous studies have explored the post-processing techniques for the AJP of copper. Lall et al. [178] thoroughly investigated process parameters such as photo sintering voltage, flash energy, pre-drying time, and temperature for printing conductive Cu lines on polyimide substrates. The ink in this work contained about 60 wt% 90 nm Cu NPs with a viscosity of about 30–40 cP. The electrical resistivity slightly increased with voltage and decreased with flash energy.

Furthermore, the samples dried at 50 °C and 65 °C exhibited similar electrical resistivity, while those pre-dried at 85 °C showed lower resistivity that further decreased with pre-drying time. These results show the importance of selecting proper sintering parameters to achieve the optimum resistivity of conductive lines for different applications. More recently, Yu et al. [179] additively manufactured nanostructured Cu and Cu-graphene composite conductive lines on ceramic substrates for electronic applications. Hydrothermally synthesized Cu nanoplates using a copper chloride precursor were dispersed in a 2 wt% hydroxy propyl methylcellulose solution in DI water. This mixture added 0.3–1.5 wt% dopamine hydrochloride to the Cu NP ink to formulate a hybrid Cu-graphene ink for conductors. The conductors printed by OPTOMEX AJ5X showed a negative temperature coefficient resistance of 0.07% °C^−1^, demonstrating the potential for high-temperature applications.

### 3.4. Challenges and Progress of Printing W

W is a refractory metal with a high melting point, good mechanical properties, and high temperature and chemical resistance. Printing this material could be beneficial for various high-temperature applications. However, solutions-based chemistry for metallic W has rarely been explored. This section will briefly overview the progress made in tungsten printing and discuss how some challenges have been addressed.

#### 3.4.1. IJP of W

Like Cu, W also has applications in electronics as a gate electrode because of its electrical conductivity and surface finishing coatings for its excellent mechanical properties such as hardness. However, printing W inks and achieving dense patterns required for optimal electrical or mechanical properties is challenging due to its high sintering temperature.

**Figure 9:**
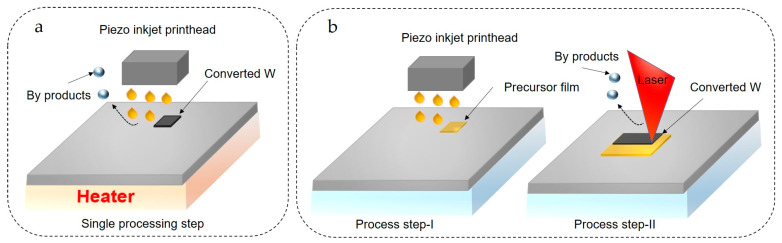
(**a**) Heat-assisted Inkjet printing (**b**) laser-assisted ink conversion with two steps; the precursor printing followed by laser conversion.

Several studies have synthesized solution-based W NPs; however, none of these works have investigated printing NP dispersions. Despite this, these studies (Table 4) provide a valuable foundation for formulating W NP-based inks for various printing techniques. To date, no articles have been published on IJP of W precursor inks. However, Gordon et al. [185] synthesized volatile liquid precursors of W for chemical vapor deposition. These liquid precursors were synthesized by complexing W(CO)_6_ with isonitriles and Lewis bases. Depending on the basicity of the Lewis bases, some were solids, while others were liquids. These new precursors could be dissolved in suitable solvents to adjust rheological properties. In doing so, these liquid precursors pave the way for IJP of W, which could then be converted to W nanostructures using a heated substrate or laser heating method (Figure 9a,b). In this case, heat-assisted IJP is analogous to the aerosol-assisted CVD method.

#### 3.4.2. DIW of W

**Figure 10:**
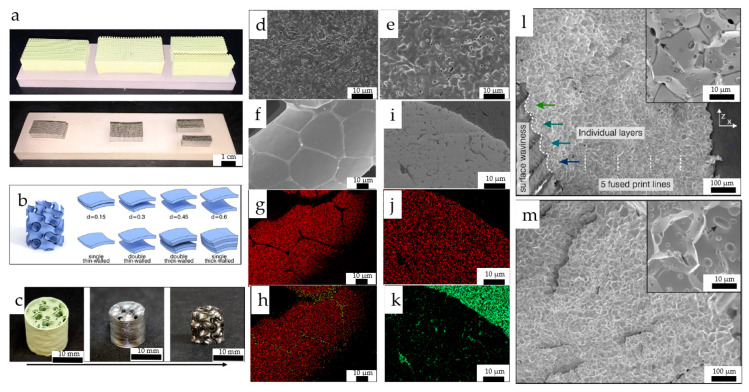
(**a**) Printed tungsten oxide micro-lattices (on top) and reduced to W (on bottom) (reproduced with permission from [180]: copyright 2018, Wiley-VCH). (**b**) different gyroid structures studied by Kenel et al.; (**c**) 3D printed structures using tungsten oxide nano-ink to the final gyroid structure after reducing and removal of the outer shell (reproduced with permission from [181]: copyright 2020, Elsevier Ltd.). (**d**,**e**) SEM micrograph of porous W printed with WO_3_ inks containing 0.5 wt% NiO without ball milling sintered at 1200 °C and 1300 °C; (**f**) SEM micrograph of porous W printed with ball-milled WO_3_ inks containing 0.5 wt% NiO sintered at 1400 °C; (**g**,**h**) EDS mapping of W in red and Ni-rich phase (in yellow) segregated at grain boundaries; (**i**) SEM micrograph of porous W printed without ball-milled WO_3_ inks containing 0.5 wt% NiO sintered at 1200 °C; (**j**,**k**) EDS mapping of W in red and Cu in green) (reproduced with permission from [180]: copyright 2018, Wiley-VCH). (**l**,**m**) SEM micrographs of the fractured gyroid wall at 20 °C and 400 °C (reproduced with permission from [181]: copyright 2020, Elsevier Ltd.).

DIW of W presents challenges due to its high sintering and melting temperatures, which are significantly higher than other materials. Dunand’s group at Northwestern University investigated DIW of W using WO_3_ NP inks [180,181]. In their first study [180], they examined the microstructure of W micro lattices (Figure 10a), Cu infiltrated composites, and additively manufactured W sheet gyroids (Figure 10b,c) in their second study [181]. The inks are developed by dispersing tungsten oxide platelets, NiO NPs in DCM, ethylene glycol butyl ether, dibutyl-phthalate dissolved in DCM, and polylactic-co-glycolic acid dissolved in DCM separately. The latter two solutions were mixed with the NP dispersion to formulate the ink, and the viscosity was optimized to 20–30 Pa·s by evaporating the solvent via ultrasonication at 50 °C. The printed 3D structures were reduced to W in an H_2_ atmosphere. The SEM micrographs in Figure 10d,e show the morphology of W struts sintered at 1200 °C and 1300 °C, indicating that porosity can be controlled by adjusting the sintering temperature. By adding NiO to the ink, high relative density parts (95% and near-full-density at 1200 °C and 1400 °C, respectively) were achieved due to the high solubility of W in Ni and the low solubility of Ni in W. In contrast, less dense parts were formed without the Ni additive. However, Ni is segregated along the grain boundaries when sintered at 1400 °C (Figure 10f–h) or at higher sintering times. The undesirable segregation of Ni reduces the strength of the W phase. They also investigated infiltrating the W structures with Cu (Figure 10i–k) to improve density by filling the voids with Cu. This material choice is interesting, as W–Cu composites can be used in thermal management applications [24,26,191]. Using these ink formulations, complex 3D W structures (cross-ply lattice and gyroid) were fabricated, and their mechanical properties were studied. Compression tests performed below and above their ductile to brittle transition temperature (20 °C and 400 °C) showed that both structures have similar stiffness, while gyroids exhibit lower peak stresses and absorption energy due to significant multiaxial stress. Figure 10l,m shows intergranular fractures at 20 °C and 400 °C for single-layer wall gyroid with high relative density.

#### 3.4.3. AJP of W

**Figure 11:**
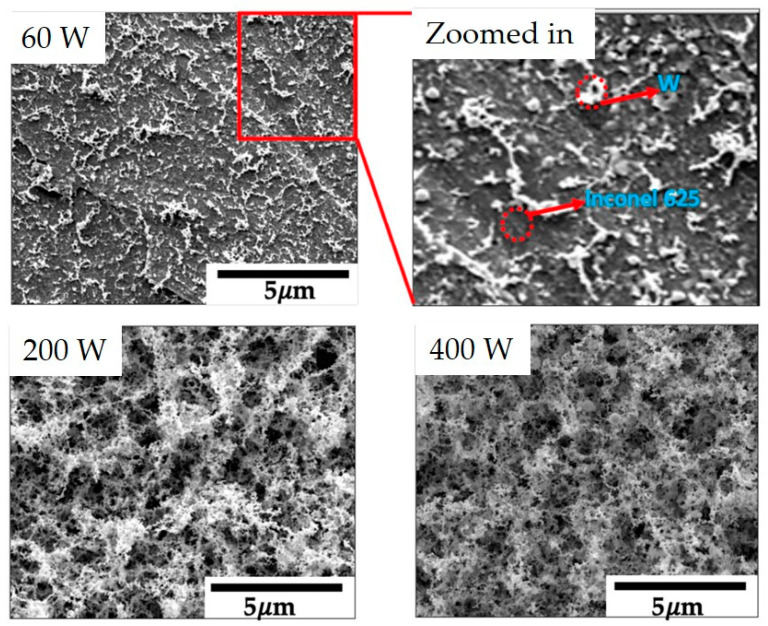
Porous networks of nanostructured W converted at different laser powers and a scanning speed of 1000 mm/s (reproduced with permission from [159]: copyright 2021, MDPI).

The AJP of W presents challenges because of the similar reasons highlighted in previous sections. The inks developed for other printing methods could be utilized for this printing technique. Interestingly, no literature is available on AJP of W except for one publication in 2021. More recently, the authors [159] demonstrated the capability of AJP, a volatile CVD precursor, then decomposing it using an IR laser for high-temperature applications. This work highlights that volatile precursors can be converted to a final product using fast heating techniques. A porous network of nanostructured W (Figure 11) was formed on Inconel 625, enhancing the base material’s solar absorptance due to the assembled nanostructures’ surface plasmon response. Furthermore, this method shows the potential to fabricate various W-based composites, and by integrating it into MAM, gradient composites can be produced. Moreover, NP inks discussed in the previous section can be used for AJP. Although producing dense tungsten would be challenging using this technique, some applications, such as catalysis and solar absorption, do not require dense structures and prefer porous ones because of high surface areas and localized surface plasmon resonance (LPSR).

## 4. Integration of SBAM Material Jetting within Powder Bed-Based MAM

This section provides a brief overview of the MAM processes integrated with SBAM technologies and then discusses the scalability of each process.

### 4.1. Binder Jetting Additive Manufacturing (BJAM)

**Figure 12:**
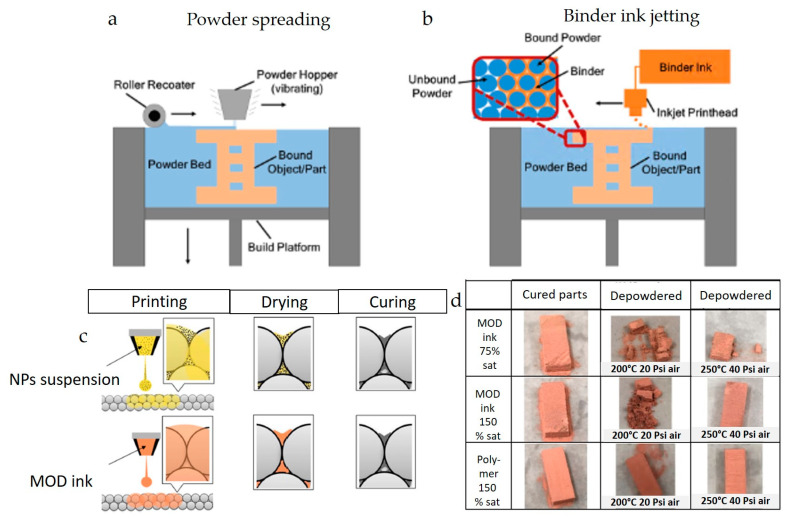
(**a**,**b**) Powder spreading in BJAM and jetting binder ink using printhead (reproduced with permission from [192]: copyright 2021, Elsevier Ltd.), (**c**) nanoparticle and particle-free precursor ink as a binder for BJAM method, (**d**) optical images of the 3D printed copper with different binder saturation ratios (reproduced with permission from [174]: copyright 2018, Elsevier Ltd.).

The inkjet printing technique is used to jet binders into the powder bed to fabricate free-form 3D objects, known as binder jetting additive manufacturing (BJAM). BJAM utilizes inkjet printing principles, in which a print head selectively applies a liquid binder to a layer of metal powder, adhering the particles to create an intermediate, or “green” part (Figure 12a,b). Afterward, the green part is subjected to a debinding process, removing the remaining binder. Finally, the part undergoes a sintering process, causing the metal particles to fuse and form a dense, solid part [192].

At Virginia Tech, researchers investigated using Cu NP dispersions and Cu MOD inks as binders to fabricate Cu parts, as illustrated in Figure 12c [174,175]. In one study [175], a 23.3 wt% Cu NP-loaded dispersion produced by Sun Chemical was employed as an NP binder in an ExOne R2 3D printer. This NP binder helped reduce organic impurities and increased the purity of the sintered part. In another study [174], a MOD precursor was synthesized using copper (II) formate, AMP, and octylamine as a particle-free binder to alleviate nozzle clogging and oxidation issues. The ink was formulated by dissolving the precursor in 2 methoxy ethanol, a common solvent for polymer binders typically used in this technique. The green parts were subsequently cured at 200 °C and 250 °C in an inert atmosphere to convert the MOD precursor to Cu before sintering the parts. Figure 12d shows the parts treated with compressed air under varying conditions. The parts with 150% saturation and a curing temperature of 250 °C were relatively stronger, although not as strong as those made with a polymer binder. This work demonstrates that particle-free inks can be used as binders; however, ink optimization with alternative solvents is necessary to enhance the bonding of powders and reduce porosity.

### 4.2. Hybrid Laser Powder Bed Fusion (LPBF)

**Figure 13:**
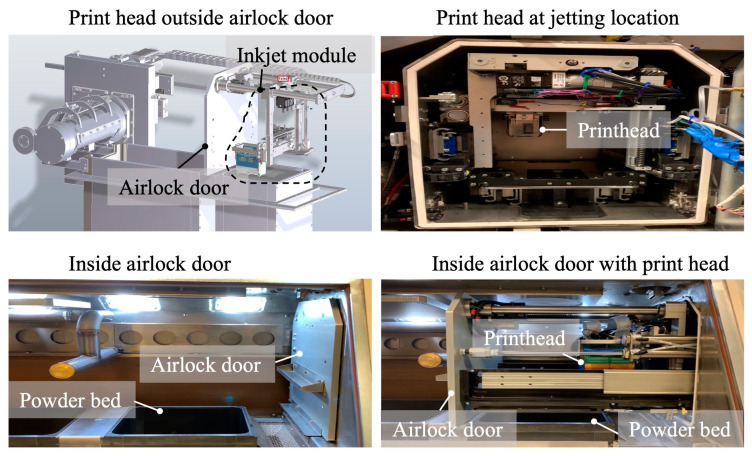
Inkjet module integrated with 3D systems LPBF machine (reproduced with permission from [194]: copyright 2020, Elsevier Ltd.).

LPBF is an AM process that uses a laser to selectively fuse a metal powder layer at the top of a powder bed, consolidating it layer by layer to produce solid parts. The process offers MAM processes the broadest range of applications [193]. Despite its widespread usage, most LPBF systems are limited to using single alloys. Paul et al. [194] at Oregon State University developed a hybrid LPBF that incorporates the inkjet printhead (XEROX M series) into a commercial LPBF (ProX DMP 300 by 3D systems) system, as shown in Figure 13. This enables doping a second-phase ink via the inkjet printhead into the powder bed prior to laser consolidation, which allows the production of metal matrix composite (MMC) or multi-material structures at a single LPBF build.

Two published papers have explored using the hybrid LPBF-Inkjet system to enhance the mechanical properties of stainless steel (SS). Oxide dispersion strengthened (ODS) 304 L SS [194] was produced using 304 L SS powder and a precursor ink made of yttrium nitrate hexahydrate Y(NO_3_)_3_ dissolved in methanol. The process involved jetting the precursor ink onto the consolidated 304 L SS, followed by laser NP synthesis and mixing, then metal powder layering and laser consolidation. ODS 316 L SS [193] was also produced by depositing an ethanol-based ink containing Al_13_ nanoclusters (NCs) onto 316 L SS powder and then processed by laser. Moreover, a 316 L SS—Cu MMC was fabricated to enhance effective thermal conductivity compared to 316 L SS by using a jettable Cu ink and emulating the hybrid LPBF-Inkjet method [48].

Like the abovementioned papers, Brigham Young University explored integrating the SBAM techniques, such as inkjet printing and direct writing, into the LPBF process [195,196]. Instead of modifying the LPBF machine to embed the SBAM modules, the feasibility of producing the MMC was demonstrated by manually doping the ink onto the powder bed using an inkjet printhead and a direct write system at each layer.

## 5. Factors Influencing Large-Scale Fabrication

Ink formulation and sintering methods are critical for scalability. These considerations are not only applicable to Cu and W but are also essential for any material that needs to be printed using material jetting techniques.

### 5.1. Scaling Up of Synthesis of NPs and Precursor for Ink

**Figure 14:**
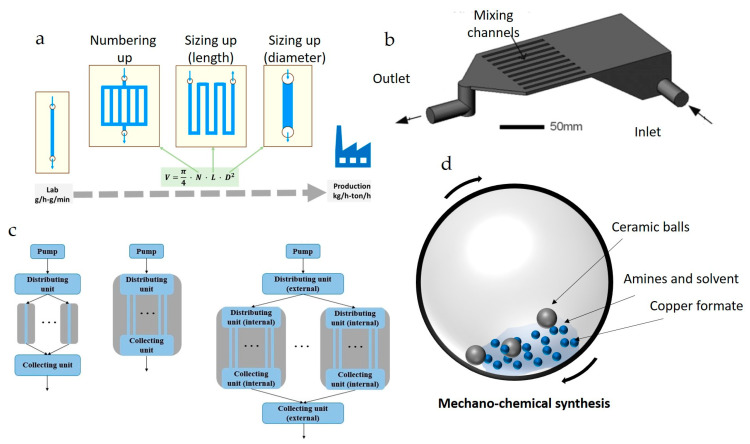
(**a**) Different scale-up approaches, (**b**) microfluidic channel numbering-up approach with minimum process control equipment, (**c**) types of flow distribution approaches (reproduced with permission from [203]: copyright 2021, Elsevier Ltd.), (**d**) mechano-chemical synthesis of copper formate-based MOD precursors with minimum solvent.

There are varying approaches to preparing ink formulations for printing methods. The first step involves synthesizing NPs or precursors, and the second step consists of dispersing or dissolving them in suitable solvents with additives. One common method is synthesizing NPs and dispersing them in solvents to create particle inks; the second method involves using precursors with additives that can influence and affect the thin film morphology and/or post-treatment of generated thin film or coatings using various heating techniques. In the former method, NPs are synthesized separately through different methods such as hydrothermal synthesis [197,198], sol-gel [199], solvothermal process [188], and electrolysis [200]. Subsequently, the NPs are washed and dispersed in solvents with surfactants and stabilizers to formulate printable inks. However, scaling-up these processes to synthesize uniformly distributed NPs and large volumes of inks can be economically challenging. Continuous flow micro/milli reactors have demonstrated promising capabilities for controlled synthesis of NPs and NPs ink [201,202]. There are many routes to scale-up from lab to industrial scale (Figure 14a), and all these routes are reviewed in detail elsewhere by Dong et al. [203]. Numbering-up approach retains the micro-scale transport phenomena and could be related to individual lab-scale synthesis. However, complex flow distribution (Figure 14b,c) needs to be investigated. These reactors could be used to produce high volumes of ink onsite, which helps avoid long-term storage of inks that can lead to precipitation, aggregation, oxidation, or other unforeseen issues.

In the latter case, direct precursors can be dissolved in solvents to formulate particle-free inks instead of using particulate inks that could clog the nozzles. When these inks are exposed to heat or light, they transform into the final products. However, precursor synthesis requires a significant amount of solvent and is not environmentally friendly. This issue can be addressed using mechanochemical synthesis (Figure 14d) [204].

The most crucial factor in both methods mentioned above is the solvent selection to adjust the ink’s rheology based on the application type, cartridge, and printhead requirements. Additionally, the choice of additives such as viscosity modifiers, surface tension modifiers, and humectants significantly impacts jetting performance.

### 5.2. Post-Processing after Printing Ink

**Figure 15:**
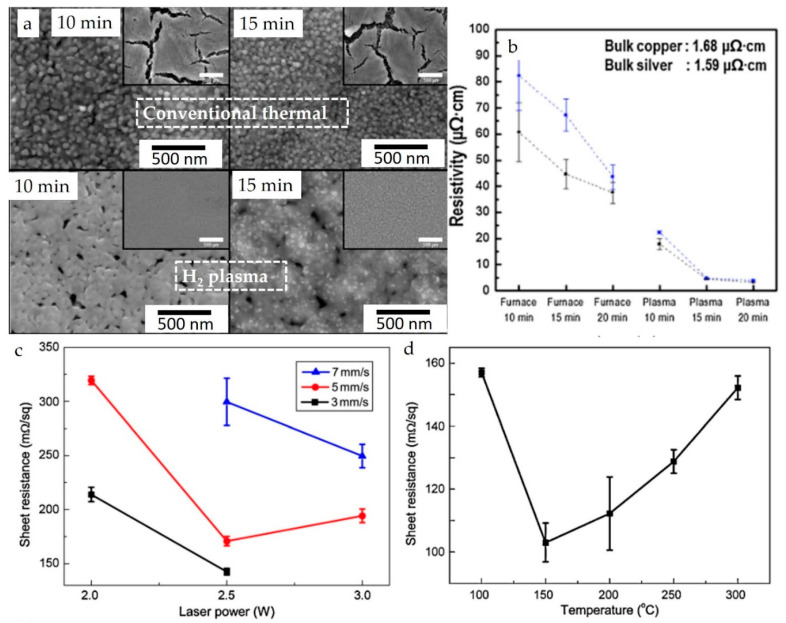
(**a**) SEM micrographs of sintered film conventionally and H_2_ plasma, (**b**) resistivities of Cu with different sintering methods at different conditions (reproduced with permission from [219]: copyright 2014, Elsevier Ltd.); (**c**) sheet resistivities of Cu using laser sintering with different laser parameters, (**d**) sheet resistivities of thermally sintered Cu at different temperatures (reproduced with permission from [220]: copyright 2014, Elsevier Ltd.).

Post-heat or pre-heat treatment is crucial for solution-based printing techniques to dry solvents, cure or remove binders and other additives, convert molecular precursors to final products [205], and sinter the printed films or structures. Various heating techniques have been explored, including conventional thermal sintering (oven or heated-bed) [206], intense pulsed light (IPL) sintering [207,208,209,210,211,212], Infrared (IR) sintering [206,213,214], microwave sintering [206,215,216], laser sintering [208,217,218], and plasma sintering [206,219]. However, additives are often necessary to enhance the absorption of radiation from non-contact heating sources to improve heating efficiency. While adding additives is not always desirable or feasible depending on several factors, these additives can sometimes enhance printed materials’ properties. For example, Kwon et al.’s investigation demonstrated that using H_2_ plasma reduced cracks in the Cu film compared to conventional thermal sintering and significantly decreased the resistivities because of enhanced densification of the films (Figure 15a,b). Figure 15c,d shows another example where laser sintering reduced the resistivities. The authors reported that this improvement was due to the enhanced microstructure of connected nanorods. Therefore, selecting efficient heating methods can reduce costs and improve the properties of the final printed materials.

### 5.3. SBAM within Powder Bed-Based MAM

#### 5.3.1. BJAM

The scalability of binder jetting makes it a promising option for industries that require high-volume production of metal parts, such as aerospace, automotive, and medical device manufacturing. This result is because a significant benefit of binder jetting, compared to other MAM processes, is its capacity to rapidly and effectively process large quantities of powder material, leading to faster production times and increased throughput. Unlike powder bed fusion (PBF), which uses a laser or electron beam to selectively melt metal powder layer by layer, binder jetting can deposit the binder material over a build area by using one or more inkjet printheads, making up an array of nozzles. The factors that affect the scalability can be as follows: (1) work envelope size; (2) number of nozzles (#); (3) printing technology; and (4) printing direction. The basic printing process steps of BJAM are (1) powder deposition; (2) powder spreading and compaction; (3) binder deposition; and (4) drying the wet binder. All BJAM systems perform similar steps with certain variations, which can influence printing speed. For example, Triple ACT technology by Desktop Metal combines the first three steps into a unified step and dries the solvent when the printing components return to the home position. Furthermore, Desktop Metal’s Single Pass Jetting (SPJ) advances the technology further by executing all process steps, including drying (no curing step), simultaneously enabling bi-directional printing. This feature significantly speeds up the printing process. Table 5 compares the maximum build rates of five selected Desktop Metal machine tools, with rates largely influenced by the previously mentioned factors. The first three machines (X-series) listed in Table 5 are designed for product development, while the last two (P-series) are intended for mass production.

#### 5.3.2. Hybrid LPBF

As outlined in Section 4.2, the hybrid LPBF-Inkjet method enables in-situ production of MMC during LPBF. Typically, powder feedstock for MMC is prepared via ball milling. However, this ball milling process poses scalability issues due to its time-consuming nature and the challenges associated with upscaling. As such, the hybrid LPBF method is expected to significantly improve scalability in MMC production by substituting ball milling with inkjet printing.

Furthermore, this hybrid system offers another advantage: the ability to create multi-material structures with spatially tailored properties at a single LPBF build. This is achievable by doping the second phase only in the desired area via the inkjet printhead. Thus, compared to conventional methods that produce multi-material structures through multiple manufacturing steps, the hybrid LPBF technique can enhance scalability by combining multiple processes into a single LPBF build.

In this hybrid method, selecting the appropriate ink is crucial. The inks require suitable viscosity, surface tension, and density to ensure stable droplet formation. Additionally, factors such as the solvent boiling point, its compatibility with the printing technology, and the concentration of solids in the inks are also important considerations when choosing the ink.

## 6. Summary and Outlook

This review article summarizes the current status of SBAM material jetting Cu and W and the integration of SBAM with powder bed-based MAM. Significant advancements have been made in various aspects of manufacturing, including types of NPs, ink formulations, post-processing techniques, and the utilization of SBAM with powder bed-based MAM. The main conclusions can be drawn as follows:

- The oxidation of Cu NPs and the printed track has been addressed through various techniques. The techniques include core-shell NPs, laser sintering, protective coating layers, and the development of new precursor MOD inks that do not require reducing agents to convert to Cu. Factors such as solvent evaporation, cooling rates, and ink composition have been reported to control the morphology and microstructure of printed parts;
- Photothermal conversion of solution-printed volatile precursors has been employed to address the challenges related to the unavailability of precursor chemistry. Additionally, wetting agents have been used to improve the sintering of W-based composites;
- The challenges associated with large-scale fabrication include synthesizing NPs or precursors for ink formulations and performing post-treatment on the printed ink. Both microfluidic channels and mechanochemical syntheses have been identified as viable approaches for scaling up ink production;
- NPs and precursor inks have been utilized to produce three-dimensional parts using BJAM, paving the way for selective doping;
- Various metal matrix composites have been fabricated using hybrid LPBF integrated with material jetting techniques;
- This hybrid LPBF method is expected to reduce manufacturing time for producing metal matrix composites by eliminating the need for mixing two different powders via ball-milling;
- This hybrid LPBF method can create functional alloys with selectively tailored thermal properties through the selective doping of Cu and W.

Despite the advantages of non-contact printing for Cu and W, the amount of research on these approaches indicates that the field is still in its infancy. The untapped aspects and critical areas of SBAM for Cu and W are outlined as follows:

- The development of Cu–Ni, Cu–Ag, and Cu–Cu formate core-shell NPs inks has been discussed in the article. However, no reported works on Cu–W or alloying Cu–W NPs with other metal NPs exist. The synthesis of such multi-alloyed NPs is possible through controlled microfluidic synthesis. Investigating the printing and sintering of these films could be beneficial, as adding W to Cu improves its mechanical properties and high-temperature resistance, while adding Cu to W enhances its electrical and thermal conductivities [26];
- Recent work by Bernasconi et al. [226] demonstrates that highly viscous fluids can be jetted using drop-on-demand piezoelectric printheads. These types of printheads could address the concern of low solid loading (percent of NPs or precursors inside the carrier solvent) in inks used in inkjet printing mechanisms for hybrid LPBF-Inkjet systems, particularly when a significant amount of second-phase material is required;
- Additionally, investigating new stand-alone W precursors or combining them with Cu MOD precursors could increase the options for selecting and formulating inks;
- Recently, grain refinement and strengthening of the Cu matrix with nanoscale W particles have been reported [227]. There are existing works on synthesizing W NPs (Table 3). Formulating inks and doping these into the powder bed could enable the fabrication of interesting three-dimensional structures for applications such as heat exchangers.

## Figures and Tables

**Table 1:** Some examples of materials printed using IJP, DIW, and AJP for various applications.

Material	Printing Method	Application	Reference
CuBi_2_O_4_	IJP	Photoelectrochemical water splitting	[100]
Pt–CB	IJP	As a catalyst to improve PEFC performance	[101]
TiO_2_	IJP	Photocatalytic degradation of pollutant	[102]
Co_3_O_4_/N-rGO	IJP	A catalyst for the oxygen reduction reaction	[103]
LaSrCoF	IJP	Catalysis	[104]
Pt/Al_2_O_3_	IJP	Catalytic reduction of NO	[105]
α-Al_2_O_3_	DIW	Catalysis	[106]
Nano Palladium	IJP	A catalyst for electroless plating	[107]
TiO_2_	DIW	Plasmonic structures for photocatalysis	[108]
Al_2_O_3_	DIW	Biomimetic porous ceramic catalyst carriers	[109]
UiO-66/polymer composites	DIW	Rapid catalytic hydrolysis of methyl paraoxon	[110]
Pd/Al_2_O_3_ CFR	DIW	Porous catalytic continuous flow reactor (CFR)	[111]
Metal Oxide/H-ZSM-5 Catalysts	DIW	A 3D-printed catalyst for hexane cracking	[112]
Silica-coated Pt/carbon	IJP	A catalyst to improve PEFCs performance	[113]
AgNO_3_/H_2_O	IJP	A catalyst for electroless plating	[114]
Ni cermet anode	IJP	The catalyst for hydrogen oxidation	[115]
3D SiC scaffold	DIW	Catalyst support for methanol steam reforming micro-reactor	[116]
Ag nanoparticles (NPs)	AJP	Wearable strain sensor	[117]
Reduced GO	AJP	3D electrodes for sensing COVID-19 antibodies	[118]
Pt	AJP	Microheaters for gas sensing applications	[119]
PEDOT: PSS/enzyme solution	IJP	Glucose sensing	[120]
Ag/AgCl/C/CNT	AJP	Electrochemical sensor for protein detection	[121]
NiO	AJP	Temperature sensor	[122]
Hydrogel	DIW	Mechanochromic sensor	[123]
WO_3_	IJP	Ultraviolet photodetectors	[124]
MOF	IJP	Ammonia gas sensor	[125]
Ag NPs	AJP	Capacitance-based strain gauge	[126]
CNT	AJP	pH sensor for live cell applications	[127]
Graphene	DIW	Gas sensing applications	[128]
Graphene	AJP	Ammonia sensing	[129]
Graphene/polyimide	IJP	Ultrasound sensors	[130]
GO/BP	IJP	Humidity sensing	[131]
IrO_x_	IJP	pH sensing	[132]
MWNT/Carbon/PDMS	IJP	Flexible deflection monitors sensing	[133]
Hydrogel electrodes	IJP	Detecting glucose, lactate, and triglycerides	[134]
PEDOT: PSS	IJP	Touch sensor	[135]
Graphene	AJP	Histamine sensor for food safety	[136]
Graphene	AJP	Immunosensor for cytokine monitoring in serum	[137]
Cu and CuNi	AJP	Flexible temperature sensors	[138]
Ru based dye	IJP	Oxygen sensing patch	[139]
Silica/NdFeB	DIW	A magnetic flexible tactile sensor	[140]
CoFe_2_O_4_	AJP	Micro supercapacitor applications	[141]
LFP, LTO/GO, CNT	DIW	Battery electrodes	[142]
LFP cathodes	AJP	High-performance cathodes	[143]
PVDF-*co*-HFP/Pyr_13_TFSI/LiTFSI/TiO_2_	DIW	Solid-state electrolyte for Li-ion batteries	[144]
Graphene	IJP	Anode for Li-ion batteries	[145]
Ni	IJP	Flexible current collector for Li-ion batteries	[146]
zinc oxide and P3HT: ICBA	IJP	Heterojunction solar cell applications	[147]
Cellulose/alginate/carbon black hydrogel	DIW	Solar steam generation	[148]
graphene	DIW	Solar steam generation	[149]
Cu (In, Ga) Se_2_	IJP	Solar absorber	[150]
α-ITO/Ag	DIW	3D Top electrodes for perovskite solar cells	[151]
Pt	AJP	Conductive tracks on polymer and ceramic substrates	[152]
Graphene	AJP	Interconnects	[153]
CNT/h-BN	AJP	1D 2D-TFTs	[154]
CNTs	AJP	TFTs	[155]
PEDOT: PSS/WO_3_/PEDOT: PSS	IJP	Flexible NVM applications	[156]
Halide perovskite-based	IJP	LEDs	[157]

**Table 3:** List of commercial copper Inks.

Manufacturer	Solvent	Post-Processing	Printing Methods	Resistivity
Applied NanoTech	-	Photo sintering	Aerosol, inkjet, and screen printing	-
Copprint	-	Hot air, IR lamp	Aerosol, flexo, gravure, inkjet, and screen printing	-
Dycotech Materials	Diethylene Glycol monoethyl ether	Laser/flash lamp, formic acid	Aerosol, inkjet, and screen printing	3.5–15 × 10^3^ µΩ □^−1^
Novacentrix	Glycol ether	Laser, formic acid vapor	Aerosol, inkjet, and screen printing	3.4–18 µΩ·cm

**Table 4:** W NPs synthesized by solution-based methods.

Method	Morphology	Size	Reference
Reverse micelle	Spherical	13 nm	[186]
Template	Nanowire	300–500 nm length and 5–8 nm dia.	[187]
Solvothermal	Spherical	15–28 nm	[188]
Sonoelectrochemistry	Spherical	30 nm	[189]
Reverse microemulsion mediated	Spherical	5 nm	[190]

**Table 5:** Comparison of BJAM system in terms of maximum build rates influenced by a few factors.

	InnoventX	X25Pro	X160Pro	P1	P50
Work envelope size (mm^3^)	165 × 65 × 65	250 × 400 × 250	500 × 800 × 400	200 × 100 × 40	490 × 380 × 260
# of Nozzles	256	2048	4096	4096	16,384
BJAM printing technology	Triple ACT	Triple ACT	Triple ACT	SPJ	SPJ
Printing direction	Uni-directional	Uni-directional	Uni-directional	Uni-directional	Bi-directional
Max build rate (cm^3^/h)	54	1200	3120	1350	12,000
Reference	[221]	[222]	[223]	[224]	[225]

## Data Availability

Not applicable.
